# Oral health related quality of life in long-term survivors of head and neck cancer compared to a general population from the seventh Tromsø study

**DOI:** 10.1186/s12903-022-02140-2

**Published:** 2022-03-30

**Authors:** Renate Andreassen, Birgitta Jönsson, Elin Hadler-Olsen

**Affiliations:** 1 Department of Otorhinolaryngology, University Hospital of North Norway, 9038 Tromsø, Norway; 2 The Public Dental Health Service Competence Center of Northern Norway, 9271 Tromsø, Norway; 3 Department of Periodontology, Institute of Odontology, The Sahlgrenska Academy at the University of Gothenburg, 41390 Gothenburg, Sweden; 4 Department of Medical Biology, Faculty of Health Sciences, UiT the Artic University of Norway, 9037 Tromsø, Norway

**Keywords:** Oral health related quality of life, Oral impact on daily performances, Head and neck cancer, General population

## Abstract

**Background:**

Both the incidence and survival rate of head and neck cancer (HNC) is increasing, making quality of life of HNC survivors an important issue.

**Methods:**

In this cross-sectional study we compared the oral health related quality of life (OHRQoL) of long-term HNC survivors to that of a general population cohort from the seventh survey of the Tromsø study with the Oral Impact on Daily Performances questionnaire. Comparisons were done with frequency analyses and cross tabulation. We also assessed OHRQoL’s association to sociodemographic and oral health related variables in both cohorts as well as with cancer related variables in the HNC cohort with regression analyses.

**Results:**

The HNC survivors had four times the risk of reporting problems with daily performances compared with the general population cohort. The ability to eat and enjoy food was most frequently affected in both cohorts. Moderate-poor self-rated dental health and general health as well as high frequency of dental visits were significantly associated with poorer OHRQoL. To have a history of oral or pharyngeal cancer was associated with more problems than having a history of laryngeal cancer.

**Conclusions:**

Our study shows that HNC treatment is associated with a strong and lasting impairment of OHRQoL, highlighting the need to find less toxic, yet effective ways to treat the disease, and to provide easy access to expert dental care at all stages of the disease to minimize morbidity. Given the widespread side effects of cancer treatment, a multidisciplinary approach might be required to improve the OHRQoL of HNC survivors.

**Supplementary Information:**

The online version contains supplementary material available at 10.1186/s12903-022-02140-2.

## Introduction

Worldwide, almost 1,000,000 persons are diagnosed with head and neck cancer (HNC) each year. The number of long-term HNC survivors is rising due to an increase in cancer incidence along with improved survival rates [1], making quality of life in this group of patients an important issue. HNC is usually treated with surgery, radiation, chemotherapy or a combination of these modalities, dependent on the tumor location, tumor stage, type of cancer and health of the patient [2]. These treatment modalities are associated with side effects, both acute and chronic. Surgery can lead to loss of important structures, scarring and deformities in a very exposed area of the body, whereas chemotherapy may cause nausea, fatigue and mucositis [3]. Radiation is associated with mucositis, permanent impaired salivary gland function, mucosal atrophy, fibrosis, trismus and reduced bone oxygenation with risk of osteoradionecrosis [4]. During the past decade, however, radiation therapy has increasingly been given as intensity-modulated radiotherapy or volumetric modulated arc therapy where the radiation dose to the healthy tissue surrounding the cancer is decreased, thereby reducing the side effects [5].

The side effects of HNC treatment may disturb several of the vital functions that the head and neck region harbors, including chewing, swallowing, speech and expression, and thus cause nutritional problems, isolation and a reduced quality of life [6]. Several studies have assessed health related quality of life (HRQoL) in long-term HNC survivors, and there seems to be a tendency that it drops during treatment, but gradually improves through the first year after treatment [7, 8]. This may be due to relief from the acute treatment side effects, such as nausea, mucositis and pain, reconstruction of lost tissue and a gradual adaptation to the chronic side effects. Most published studies use quality of life assessment tools that are specifically developed for HNC cancer patients such as the EORTC QLQ-C30 together with the QLQ-H&N35 [9, 10]. Several studies have also assessed the more specific oral health related quality of life (OHRQoL) of HNC patients, and find that it is severely affected during treatment and the first year after treatment [11–13]. However, few studies have assessed the OHRQoL in long-term HNC survivors and compared it to a general population, to see whether it reverts towards the general population with time.

There are several validated questionnaires to evaluate OHRQoL, including the Oral Impact on Daily Performances (OIDP) [14]. This questionnaire assesses how often during the past 6 months problems with the mouth or the teeth have caused various functional, psychological and social problems. The tool has been used widely [15], and therefore allows comparison of the OHRQoL between populations or subgroups with extraordinary challenges related to oral functions. Knowledge of OHRQoL may help both clinicians and health authorities to evaluate treatment need and to prioritize between groups of patients, and it may be a tool to evaluate the effect of interventions. The aim of the present study, was to assess OHRQoL in a cohort of patients treated for HNC as well as in general adult population in Norway, by use of the OIDP questionnaire. We also aimed at determining the associations between OHRQoL and sociodemographic factors, general health, dental health and use of dental services in both cohorts, as well as with cancer treatment, cancer location and year of cancer diagnosis in the HNC cohort.

## Methods

This cross-sectional study aimed at assessing OHRQoL and associated factors in HNC survivors and in a general adult population in Norway. The study included two separate cohorts, one with persons treated for HNC (HNC cohort) and one general, adult population from the seventh survey of the Tromsø study (T7 cohort), which is a community-based cohort study. The characteristics of both cohorts are summarized in Table 1. The study was conducted in accordance with the Declaration of Helsinki with informed consent from the participants, and the Regional Committee for Health Research approved it (REK79888). The study was conducted and reported in accordance with the STROBE checklist for observational studies [16].

**Table 1:** Cohort characteristics

	HNC cohort n (%)	T7 cohort n (%)	p*
*Gender*			
Male	126 (58.3)	6045 (47.8)	0.002
Female	90 (41.7)	6602 (52.2)	
*Age group*			
< 60 years	38 (17.6)	5552 (43.9)	< 0.001
60–69 years	82 (38.0)	4461 (35.3)	
≥ 70 years	96 (44.4)	2634 (20.8)	
*Education*			
No university degree	126 (58.3)	7017 (56.5)	0.599
University degree	90 (41.7)	5393 (43.5)	
*General health*			
Good	103 (47.7)	8552 (68.2)	< 0.001
Moderate	82 (38.0)	3343 (26.6)	
Poor	31 (14.4)	653 (5.2)	
*BMI*			
Underweight < 18.5	8 (3.8)	75 (0.6)	> 0.001
Normal 18.5–24.99	103 (48.8)	3915 (31.1)	
Overweight ≥ 25	100 (47.4)	8618 (68.4)	
*Dental health*			
Good	91 (42.19)	6521 (52.8)	< 0.001
Moderate	65 (30.2)	4557 (36.9)	
Poor	59 (27.4)	1282 (10.4)	
*Dental visits*			
> 1/year	152 (70.7)	3097 (25.1)	< 0.001
1/year	40 (18.6)	6520 (52.8)	
1/2nd year	6 (2.8)	942 (7.6)	
< 1/2nd year	17 (7.9)	1796 (14.5)	
*Time of cancer diagnosis*			
Before 2010	87 (40.3)		
2010–2014	68 (31.5)		
2015–2020	61 (28.2)		
*Cancer location*			
Oral cavity	60 (27.8)		
Pharynx	98 (45.4)		
Hypopharynx/Larynx	52 (24.1)		
Other/unspecified	6 (2.8)		
*Cancer treatment*			
Surgery, no radiation	17 (7.9)		
Radiation^a^	22 (10.2)		
Radiation + surgery^b^	176 (81.5)		
*Recurrence*			
No	167 (77.7)		
Yes	48 (22.3)		

*Statistical significance assessed by the Pearson’s chi-square test,

^a^Radiation with or without adjuvant chemotherapy

^b^Radiation and surgery with or without adjuvant chemotherapy

### HNC cohort

In May 2020, a questionnaire was sent by mail to the 577 members of the “Head and neck cancer association”. This is an association that works for the interests of HNC patients in Norway, and have members who have or have had cancer in the head and neck region themselves, their next of kin, as well as professionals working with HNC. One reminder was sent by SMS, and the last response was received by the end of August 2020. Responses from members without a history of HNC (next of kin or health care professionals working with HNC) were excluded.

The questionnaire assessed sociodemographic variables, general health and oral health related variables by the same questions as in the T7 cohort, but also included questions associated with the cancer diagnosis and treatment.

### T7 cohort

The seventh survey of the Tromsø Study was carried out in 2015 and 2016. An invitation was sent by mail to all 32,591 persons 40 years or older in Tromsø municipality. In the present study, we used data from a questionnaire in T7 assessing sociodemographic variables, general health and oral health related variables. We excluded persons who had reported a current or previous cancer diagnosis (n = 1636, 7.8%). Further, we excluded persons younger than 50 years of age (n = 6432, 33%) to increase the match with the HNC cohort, which included only five persons younger than 50 years.

### Variables

The following variables were included from both cohorts:

#### Outcome variable

We assessed OHRQoL by the Norwegian version of the OIDP questionnaire [17], where the respondents rate how often during the past 6 months problems with their teeth or mouth have caused difficulties in eight areas of their life (Table 2). There are five response options: 0: never; 1: less than monthly; 2: once or twice a month; 3: once or twice a week; and 4: every or almost every day. For analyses, we dichotomized OIDP into no problems (option 0) versus problems (options 1–4) for each of the eight questions as well as for all questions combined. This is a common way of analyzing this questionnaire [17–20]. Cronbach’s Alpha for OIDP was 0.82 for the HNC cohort and 0.93 for the T7 cohort.

**Table 2:** Oral impact on daily performances (OIDP) for the HNC and the T7 cohorts

Daily performance	HNC cohort n (%)	T7 cohort n (%)	p*
*OIDP total*			
No problems	34 (16.4)	9683 (80.5)	< 0.001
Problems	173 (83.6)	2341 (19.5)	
*Eat and enjoy food*			
No problem	66 (31.4)	10,685 (87.0)	< 0.001
Problem	144 (68.6)	1594 (13.0)	
*Speak and pronounce*			
No problem	91 (44.8)	11,677 (95.4)	< 0.001
Problem	112 (55.2)	565 (4.6)	
*Clean teeth*			
No problem	132 (64.7)	11,315 (92.5)	< 0.001
Problem	72 (35.3)	913 (7.5)	
*Smile/show teeth*			
No problem	145 (70.7)	11,140 (91.2)	< 0.001
Problem	60 (29.3)	1080 (8.8)	
*Sleep and relax*			
No problem	114 (56.2)	11,576 (94.8)	< 0.001
Problem	89 (43.8)	637 (5.2)	
*Emotionally stabile*			
No problem	110 (53.9)	11,553 (94.7)	< 0.001
Problem	94 (46.1)	652 (5.3)	
*Enjoy company*			
No problem	122 (59.5)	11,428 (93.7)	< 0.001
Problem	83 (40.5)	762 (6.3)	
*Daily tasks*			
No problem	122 (60.1)	11,770 (96.9)	< 0.001
Problem	81 (39.9)	377 (3.1)	

*Statistical significance assessed by the Pearson’s chi-square test

#### Explanatory variables

Age was trichotomized into (1) 50–59 years, (2) 60–69 years and (3) 70 years or older. In the HNC cohort, there were five respondents (1.9%) younger than 50 years, and they were included in the 50–59-year group. Gender had the response options man or woman.

Highest level of education was dichotomized into (1) no college/university degree (including the original options ≤ 10 years of school and high school) and (2) college/university degree (including college/university degree ≤ 3 years and college/university > 3 years).

Self-rated general health and self-rated dental health was assessed by one question each with five response options from 1 (very poor) to 5 (very good). For analyses, we trichotomized the responses into: (1) poor (option 1 and 2); (2) moderate (option 3); and (3) good (option 4 and 5). Dental attendance was assessed with one question with six options. For regression analyses, we trichotomized the options into (1) more often than once a year; (2) once a year; and (3) every second year or rarer. We calculated the body mass index (BMI = weight in kilograms divided by the square of height in meters), and trichotomized it into (1) underweight (< 18.5); (2) normal weight (18.5–24.99), and (3) overweight (≥ 25).

For the HNC cohort we also included cancer-associated variables:

Cancer location in the head and neck region according to the WHO classification were listed in the questionnaire, and the respondents checked all relevant location. For analyses the locations were categorized as (1) oral cavity, (2) pharynx (including oropharynx and nasopharynx) and (3) larynx (including hypopharynx and larynx) according to the 4th edition of the WHO classification of head- and neck tumors [21]. Nasopharyngeal and oropharyngeal cancers were grouped due to a low number of nasopharyngeal cancers (n = 4).Three people reported salivary gland cancer and one reported cancer in the maxillary sinuses, they were all included in the oral cavity cancer group because of the close anatomical proximity. Cancers located in both the oral cavity and the pharynx were coded as oral cavity, whereas cancers involving both the pharynx and the larynx were coded as pharyngeal cancers.

We assessed cancer treatment with one question with 5 options: (1) surgery; (2) radiation; (3) chemotherapy; (4) other (with free-text option) and (5) do not know, and instructed the respondents to check all relevant options. We re-categorized cancer treatment as (1) surgery without radiation, (2) radiation only and (3) radiation and surgery with or without adjuvant chemotherapy. No respondents had received chemotherapy only.

Time of first cancer diagnosis was categorized as (1) before 2010; (2) 2010–2014; and (3) 2015–2020. Cancer recurrence was reported as yes or no.

### Statistical analyses

We used the Statistical package for Social Sciences (SPSS) for Windows version 26 (IBM corporation, Armonk, NY, USA) for statistical analyses. We assessed differences among groups by cross-tabulation, and the statistical significance of the observed differences were assessed by Pearson’s Chi-square test. In relative risk analyses by cross-tabulation as well as in univariate and multivariate logistic regression analyses (forward, stepwise) we used OHRQoL dichotomized into no problems versus problems as the dependent variable, with no problem as the reference value. The variables listed in Table 1 were used as explanatory variables in regression analyses, but BMI was excluded due to the low number of underweight persons. We present the results from the relative risk analyses as relative risk (RR) with 95% confidence intervals (95% CI) and from regression analyses as odds ratios (OR) with 95% CI. The significance level is set to *p* < 0.05 for all analyses. Data were missing for less than 5% of the variables included in regression analyses, and these were excluded from analyses.

## Results

We received 349 (59%) answers from the HNC cohort, of which 216 were from members with a history of HNC themselves. The remaining 133 responders were either next of kin to HNC patients or health care professionals working with HNC, whom we excluded. The attendance rate of the T7 study was 65% (N = 21,083), of whom 12,647 were included in this study after exclusion of respondents with a current or previous cancer diagnosis or age younger than 50 years.

Table 1 summarizes the characteristics of the two cohorts.

Compared to the T7 cohort, the HNC cohort included a higher proportion of men and people of older age, whereas educational level was similar. The HNC survivors reported significantly worse general health and lower BMI than did respondents in the T7 cohort. Furthermore, the HNC survivors had poorer self-rated dental health than respondents in the T7 cohort had, and reported a much higher frequency of dental visits with 71% having regular visits more than once a year, compared to 25% in the T7 cohort. The most common cancer location was the pharynx, and more than 90% of the HNC survivors had been treated with radiation therapy (Table 1). Half of the patients extracted teeth in association with the treatment. About 50% of the patients with oral cancer and almost 10% of the patients with pharyngeal cancer had part of their tongue excised during surgery, whereas 50% of those with laryngeal cancers had tracheostomies (Additional file 1: Table S1).

Table 2 summarizes the OIDP scores for the two cohorts, combined and for each of the eight activities.

For each of the eight daily activities assessed by the OIDP instrument, respondents in the HNC cohort reported a much higher frequency of problems than did those of the T7 cohort, with only 16% of the respondents reporting no problems with any of the activities, compared to 81% in the T7 cohort. The daily activity that was most frequently affected in both cohorts was the ability to eat and enjoy food, with which more than two thirds reported problems in the HNC cohort and 13% in the T7 cohort. The ability to speak and pronounce words clearly was the second most affected daily performance in the HNC cohort, with which more than half of the respondents reported problems. The ability to smile and show teeth without embarrassment was the second most affected daily performance in the T7 cohort, but the least affected in the HNC cohort. Still, a much higher proportion of the HNC cohort than of the T7 cohort reported problems with smiling or showing teeth (29% vs 9%, Table 2).

As the HNC- and the T7 cohorts were unmatched for age and gender, we performed a cross tabulation of OIDP frequency score and these variables, which confirmed that the HNC cohort had much higher OIDP scores than the T7 cohort had, across gender and age (Additional file 1: Table S2). Cross-tabulation analyses showed that HNC survivors had a relative risk of 4.02 of reporting problems compared to persons in the T7 cohort (95% CI 3.76–4.30).

To explore how the panorama of problems varied with tumor location and treatment, we performed cross-tabulation of OIDP with tumor location (Table 3) as well as with cancer treatment (Table 4). This revealed that tumors in the oral cavity and the pharynx were associated with over-all more severe impact on daily performances than laryngeal tumors (91%, 88% and 64% reporting problems respectively). Compared to respondents with a history of laryngeal cancer, a significantly higher proportion of those with a history of oral cavity or pharyngeal cancer reported problems with eating or enjoying food. Furthermore, those with a history of oral cavity cancer had a much higher fraction reporting speech problems than those with a history of pharyngeal or laryngeal cancer (Table 3).

**Table 3:** Oral impact on daily performance by cancer location

Daily performance	Oral cavity n (%)	Pharynx n (%)	Larynx n (%)	p*
*OIDP total*				
No problems	5 (8.6)	12 (12.5)	17 (36.2)	< 0.001
Problems	53 (91.4)	84 (87.5)	30 (63.8)	
*Eat and enjoy food*				
No problem	13 (22.4)	23 (24.2)	29 (56.9)	< 0.001
Problem	45 (77.6)	72 (75.8)	22 (43.1)	
*Speak and pronounce*				
No problem	17 (29.3)	46 (48.9)	25 (55.6)	0.015
Problem	41 (70.7)	48 (51.1)	20 (44.4)	
*Clean teeth*				
No problem	32 (56.1)	62 (67.4)	35 (71.4)	0.213
Problem	25 (43.9)	30 (32.6)	14 (28.6)	
*Smile/show teeth*				
No problem	34 (60.7)	70 (74.5)	37 (75.5)	0.142
Problem	22 (39.3)	24 (25.5)	12 (24.5)	
*Sleep and relax*				
No problem	29 (50.9)	48 (51.6)	34 (72.3)	0.040
Problem	28 (49.1)	45 (48.4)	13 (27.7)	
*Emotionally stabile*				
No problem	28 (50.0)	52 (54.7)	28 (59.6)	0.623
Problem	28 (50.0)	43 (45.3)	19 (40.4)	
*Enjoy company*				
No problem	33 (57.9)	57 (60.0)	31 (66.0)	0.687
Problem	24 (42.1)	38 (40.0)	16 (34.0)	
*Daily tasks*				
No problem	33 (58.9)	56 (59.6)	31 (66.0)	0.717
Problem	23 (41.1)	38 (40.4)	16 (34.0)	

*Statistical significance assessed by the Pearson’s chi-square test

**Table 4:** Oral impact on daily performances by cancer treatment

Daily performance	Surgery no radiation n (%)	Radiation^a^ n (%)	Radiation and surgery^b^ n (%)	p*
*OIDP total*				
No problems	7 (41.2)	4 (20.0)	23 (15.2)	0.030
Problems	10 (58.8)	16 (80.0)	128 (84.8)	
*Eat and enjoy food*				
No problem	11 (64.7)	6 (28.6)	48 (28.9)	0.010
Problem	6 (35.3)	15 (71.4)	118 (71.1)	
*Speak and pronounce*				
No problem	12 (70.6)	10 (52.6)	66 (41.0)	0.050
Problem	5 (29.4)	9 (47.4)	95 (59.0)	
*Clean teeth*				
No problem	14 (82.4)	14 (70.0)	101 (62.7)	0.242
Problem	3 (17.6)	6 (30.0)	60 (37.3)	
*Smile/show teeth*				
No problem	12 (70.6)	19 (95.0)	110 (67.9)	0.042
Problem	5 (29.4)	1 (5.0)	52 (32.1)	
*Sleep and relax*				
No problem	16 (94.1)	12 (60.0)	83 (51.9)	0.004
Problem	1 (5.9)	8 (40.0)	77 (48.1)	
*Emotionally stabile*				
No problem	13 (76.5)	14 (70.0)	81 (50.3)	0.041
Problem	4 (23.5)	6 (30.0)	80 (49.7)	
*Enjoy company*				
No problem	14 (82.4)	14 (70.0)	93 (57.4)	0.090
Problem	3 (17.6)	6 (30.0)	69 (42.6)	
*Daily tasks*				
No problem	14 (82.4)	13 (65.0)	93 (58.1)	0.139
Problem	3 (17.6)	7 (35.0)	67 (41.9)	

^a^Radiation with or without adjuvant chemotherapy

^b^Radiation and surgery with or without adjuvant chemotherapy

*Statistical significance assessed by the Pearson’s chi-square test

Respondents who were treated without radiation therapy generally reported better OHRQoL than those who had received radiation, with poorest OHRQoL among respondents who had received radiation in combination with surgery and/or chemotherapy (Table 4). Respondents who were treated with radiation, either alone or in combination with other treatment, reported significantly more problems with eating and enjoying food, with sleeping and relaxing, and with being emotionally stabile than those who had received surgery only. Respondents who had received only radiation therapy reported less problems with smiling or showing teeth without embarrassment than did those receiving surgery or radiation in combination with surgery and/or chemotherapy (Table 4).

To assess the associations between the outcome variable OIDP and the explanatory variables, we performed regression analyses for both cohorts, with OIDP dichotomized into no problems and problems (Table 5). For the HNC cohort, we ran two multivariate models, one where we corrected for the same variables as for the T7 cohort (partly adjusted model), and one where we additionally included the cancer specific variables (fully adjusted model).

**Table 5:** Regression analyses for oral impact on daily performance

	HN cohort No problems vs. problems			T7 cohort No problems vs. problems	
	Unadjusted OR (95% CI)	Adjusted OR (95% CI) Reduced model	Adjusted OR (95% CI) Full model	Unadjusted OR (95% CI)	Adjusted OR (95% CI)
*Gender*					
Male	1.00			1.00	
Female	1.18 (0.55–2.51)			**0.87 (0.81–0.93)**	
*Age group*					
< 60 years	**4.85 (1.05–22.22)**			**1.25 (1.11–1.42)**	**1.65 (1.43–1.90)**
60–69 years	0.70 (0.32**–**1.51)			**1.15 (1.01–1.31)**	**1.27 (1.10–1.47)**
≥ 70 years	1.00			1.00	1.00
*Education*					
No university degree	0.98 (0.47–2.07)			1.01 (0.94–1.09)	**0.84 (0.76–0.93)**
University degree	1.00			1.00	1.00
*General health*					
Good	1.00	1.00	1.00	1.00	1.00
Moderate	**4.75 (1.85–12.20)**	**3.54 (1.29–9.69)**	**4.28 (1.43–12.83)**	**1.60 (1.48–1.74)**	**1.18 (1.05–1.32)**
Poor	**10.27 (1.33–77.32)**	6.28 (0.75–2.33)	6.02 (0.68–53.45)	**2.67 (2.31–3.08)**	**1.81 (1.48–2.22)**
*Dental health*					
Good	1.00	1.00	1.00	1.00	1.00
Moderate	2.26 (0.96–5.31)	1.93 (0.74–5.00)	2.60 (0.91–7.48)	**3.12 (2.86–3.39)**	**2.84 (2.54–3.18)**
Poor	**10.75 (2.42–47.65)**	**6.78 (1.44–31.92)**	**9.04 (1.77–46.13)**	**12.54 (11.14–14.10)**	**11.42 (9.79–13.33)**
*Dental visits*					
> 1/year	1.00	1.00	1.00	1.00	1.00
1/year	**0.21 (0.09–0.47)**	**0.23 (0.09–0.56)**	**0.29 (0.11–0.77)**	**0.52 (0.47–0.57)**	**0.65 (0.58–0.74)**
≤ 1/2nd year	0.74 (0.20–2.81)	0.58 (0.13–2.54)	1.10 (0.21–5.82)	1.02 (0.92–1.12)	**0.84 (0.73–0.96)**
*Diagnosis*					
Before 2010	1.00				
2010–2014	1.58 (0.67–3.70)				
2015–2020	2.35 (0.87–6.36)				
*Cancer location*					
*Oral cavity*	**6.01 (2.01–17.92)**		**7.19 (2.00–25.83)**		
Pharynx	**3.97 (1.70–9.27)**		**6.22 (2.13–18.16)**		
Hypopharynx/Larynx	1.00		1.00		
*Cancer treatment*					
*Surgery*	1.00				
Radiation	2.80 (0.65–12.06)				
Radiation +	**4.47 (1.55–12.93)**				
*Recurrence*					
*No*	1.00				
Yes	0.95 (0.40–2.27)				
*Model summary*					
Nagelkerke R-square		R^2^ = 0.29	R^2^ = 0.38		R^2^ = 0.18
Hosmer and Lemeshow test		*P* = 0.798	*P* = 0.425		*P* = 0.521
Omnibus Test		*P* < 0.001	*P* < 0.001		*P* < 0.001

Numbers in bold indicate statistically significant associations

Irrespective of cohort and model, self-rated dental health showed the strongest association with OIDP. In the fully adjusted model for the HNC cohort, those who rated their dental health as poor had nine times the odds of reporting problems with one or several daily activities compared to those rating their dental health as good. The association was even stronger in the T7 cohort with an odds ratio of eleven. Having poor or moderate self-rated general health was also associated with reporting problems with daily performances in both cohorts, as was having dental visits more than once a year. In the adjusted model for the T7 cohort, lower age and higher educational level also increased the odds of reporting problems with daily performances. In the fully adjusted model for the HNC cohort, cancer location in the oral cavity or the pharynx was strongly associated with reporting problems, in addition to the significant variables from the partly adjusted model. The fully adjusted model for the HNC cohort explained almost 40% of the variance in OIDP, whereas the partly adjusted model explained almost 30%. For the T7 cohort, the adjusted model explained almost 20% of the variance.

## Discussion

In the current study, we have assessed OHRQoL by the OIDP questionnaire in a cohort of patients treated for HNC and in a general adult population cohort in Norway. Respondents in the HNC cohort had about 4 times the relative risk of reporting problems with oral functions compared with those in the general population cohort. The HNC survivors reported most problems with eating and speech, but problems related to emotional and social functioning were also common. Moderate-to-poor self-rated dental health and general health as well as high frequency of dental visits were all significantly associated with poorer OHRQoL in both cohorts. HNC survivors who had a history of oral or pharyngeal cancer had more problems than those with a history of laryngeal cancer.

The long-term side effects of HNC treatment, especially related to radiation therapy, are well documented [4]. It is therefore not surprising that the OIDP scores for the HNC cohort were higher than for the general population, but the extent of the difference is worrying. More than 80% of the HNC survivors experienced problems with at least one daily performance, which shows that having a history of HNC is associated with a lasting, poor OHRQoL. Very few studies have compared OHRQoL in long-term HNC survivors to a general population. One previous Spanish study assessed OHRQoL in long-term survivors of oral cancer and a gender and age-group matched control population and found that the cancer patients had about 20 times higher odds for having problems with daily functions, assessed by the OIDP questionnaire [22].

We found that the proportion reporting problems was somewhat lower among head and neck cancer survivors who were diagnosed a long time before the survey compared to those diagnosed within the past 10 years of the survey. However, the association between time of diagnosis and OHRQoL was not statistically significant, neither in univariate nor in multivariate regression analyses. The majority of those who were diagnosed 0–10 years prior to the survey would have been treated with intensity-modulated radiotherapy or volumetric modulated arc therapy. Although these treatments reduce the radiation dose to the healthy tissue surrounding the cancer [23], many of those who were treated after the introduction of these refined radiation methods still reported a poor OHRQoL. This is in agreement with a previous study finding that dysphagia, which is associated with eating problems, is reduced with lower radiation doses to salivary glands, but still a significant problem after intensity-modulated radiotherapy [24]. In cross-tabulation analyses we found that those who had received radiotherapy reported significantly more problems with daily activities over-all than those who were treated without radiation, which is in line with findings from a review of papers assessing HRQoL and OHRQoL in HNC patients [25]. On the other hand, we found that esthetical problems were most prevalent among HNC survivors who had received surgery alone or in combination with radiotherapy, which may be due to scarring or deformities. Type of cancer therapy was not significantly associated with OHRQoL in multivariate regression analyses, which is probably due to the low number of respondents who had been treated with mono therapy (surgery or radiation only) in the HNC cohort. This cause unbalanced group sizes and low statistical power to the analyses. Nevertheless, our study shows that OHRQoL is poor also among long-term HNC survivors who have not received radiotherapy. This is in line with a previous study, which assessed OHRQoL 1 and 6 months after different types of HNC therapy [13].

Regular dental visits have been associated with good dental health and OHRQoL in many studies [26–29]. This is probably because of the high frequency of dental diseases such as caries and periodontitis that have no or subtle symptoms in early phases [30]. Regular dental visits allow disease prevention or detection and treatment before the disease reaches an advanced stage. Thus, our finding that regular dental visits more than once a year was associated with poorer OHRQoL than yearly visits in both the HNC and the T7 cohort was somewhat surprising. Studies often categorize dental visiting pattern into routine attenders and problem-oriented attenders [31], and usually merge those having regular dental visits yearly and those having more frequent dental visits. However, our study suggests that those who have very frequent dental visits define a group with particular oral health related problems also in the general population, and they should maybe be categorized as problem-oriented attenders, although they have regular visits. This may be problems that require recurrent treatment, but where the dentist or dental hygienist cannot cure or eradicate the problem or disease. There is no cure for many of the common side-effects of HNC treatment, such the xerostomia, but regular dental treatment may be required to prevent or treat caries or periodontitis that can follow the xerostomia, and to restore or replace teeth damaged or lost due to the cancer treatment. Our study gives no information about the type of dental care that the patients have received. Recently, guidelines for how to diagnose, treat and follow up HNC in Norway were published by the Norwegian health authorities [32], which state that HNC patients should be closely followed up by a dentist the first year after cancer treatment. However, no suggestions on how to best alleviate the oral problems are included in the guidelines. Our study shows that the need for oral health care extends much farther than the first year after cancer treatment, and the health service plan should give advices on how to alleviate oral problems. Some studies have shown a positive effect on quality of life parameters of interventions giving counselling based on HNC patients concerns and problems [33, 34]. Such patient concern inventories could be tested in a systematic manner also in Norway, maybe in association with the frequent dental visits, as our study strongly suggests a need to develop better strategies for treating or limiting the oral side effects of HNC treatment.

We found that OHRQoL was significantly associated with general health in both study cohorts, which is in accordance with findings from several other studies [35–37]. Oral health and general health can affect each other mutually. Poor general health may negatively affect a person’s ability to take care of the oral health, such as to clean the teeth or to seek dental treatment. In turn, oral pathogens can be inhaled and cause pulmonary disease [38]. Furthermore, poor oral health may affect eating habits, and thereby cause nutritional problems [39–41]. To eat and enjoy food was the daily activity most frequently affected in both cohorts in the current study, but with a much higher proportion reporting problems in the HNC cohort compared to the T7 cohort. Accordingly, we found a higher BMI among respondents in the T7 cohort than in the HNC cohort, with 68% and 47% being overweight, respectively. Although few had a BMI corresponding to underweight, almost a quarter of the respondents in the HNC cohort had a BMI in the lower range of normal weight, below 22. For elderly persons, several studies have found that having a BMI in the higher range of normal weight, or even being overweight is associated with better functional level and nutritional status [42, 43]. The majority of the HNC cohort were 70 years or older, which suggests that many of them might have experienced general health benefits from gaining weight. A previous study has also found that oral cancer patients often are at risk of malnutrition [44]. Therefore, professional guidance on how to ensure adequate nutritional status despite eating problems could improve both the general health and the OHRQoL of HNC survivors. To do this effectively, it would be useful to increase the understanding of the nature of the eating problems including their association with dental status, xerostomia, taste disturbances, trismus and dysphagia.

There are several limitations to this study. It is based on self-reported measures, relying on the memory of the respondents. This limits the information about clinical parameters such as tumor stage at diagnosis and details regarding therapy including type of surgery and radiation protocol and radiation field. We did not include such questions in the study as we considered it unlikely that the study participants had accurate knowledge of this. Furthermore, we lack clinical data on dental status and include only self-rated dental health as a measure of the dental status of the participants. In the T7 cohort respondents with a cancer diagnosis were excluded, and one could therefor argue that it is not a proper general population. Our study may therefore overestimate the OHRQoL of the actual general population. However, a previous study using OIDP to measure OHRQoL in Norwegian adults found that about 18% of adults between 45 and 79 years reported problems with at least one daily performance [45], which is close to, but slightly lower than our findings.

The number of respondents in the two cohorts was very different, and this will affect the power of the statistical analyses, which is much higher for the T7 cohort than for the HNC cohort. This may explain why fewer associations were statistically significant in regression analyses for the HNC cohort than for the T7 cohort, as well as the range of the 95% CI of the OR. Furthermore, the HNC cohort is probably not representative for all persons with a history of HNC in Norway. We surveyed members of the organization working for HNC patients in Norway. The rationale for selecting members of this organization was to avoid contacting persons with a HNC cancer history who did not want to be reminded of their illness, because that may be stressful [46]. As the patient organization aims at providing information to their members on how to handle life after a HNC diagnosis, the members may be better informed of their rights and how to best handle their oral health problems than the average HNC survivor. Also, it is to be expected that the frailest of the members of the HNC patient organization were less likely to respond to the questionnaire than the healthier ones. Thus, we believe that our survey is more likely to overestimate the OHRQoL of the general HNC survivor in Norway than to underestimate it. This is supported by findings from a study assessing late effects and long-term quality of life in HNC cancer patients participating or declining to participate in a controlled intervention study. Those who declined had poorer functional level and quality of life as well as more severe symptoms than those who participated [47].

## Conclusions

Our study shows that HNC survivors have a strong and lasting impairment of OHRQoL, highlighting the need to find less toxic, yet effective ways to treat the disease. Tight follow-up of dental professionals does not seem to alleviate the problems. Given the widespread negative effects associated with cancer treatment, a multidisciplinary approach including dental hygienists, general dentists, various dental specialists, nutritional specialists, speech therapists and psychologists might be required to improve the OHRQoL in HNC survivors.

## Supplementary Information

**Additional file 1.** **Table S1.** Additional treatment information Head and neck cancer cohort. **Table S2.** Oral impact on daily performances by age and gender.

## Data Availability

The data that support the findings of this study are available from Tromsøundersøkelsen but restrictions apply to the availability of these data, which were used under license for the current study, and so are not publicly available. Data are however available from the authors upon reasonable request and with permission of Tromsøundersøkelsen. Data from the HNC cohort are not publicly available due to privacy and ethical regulations but are available from the corresponding author on reasonable request.

## Abbreviations

HNC: Head and neck cancer
OHRQoL: Oral health related quality of life
HRQoL: Health related quality of life
OIDP: Oral Impact on Daily Performances
T7 cohort: Adult population from the seventh survey of the Tromsø study
BMI: Body mass index
OR: Odds ratios
CI: Confidence interval
