# Supplementary material for: Oral health related quality of life in long-term survivors of head and neck cancer compared to a general population from the seventh Tromsø study
Source: BMC Oral Health. 2022 Mar 30;22:100. doi: 10.1186/s12903-022-02140-2 (PMC8969380; doi:10.1186/s12903-022-02140-2)
Supplement: Supplementary file 1 — Additional file 1. Table S1. Additional treatment information Head and neck cancer cohort. Table S2. Oral impact on daily performances by age and gender. [file 12903_2022_2140_MOESM1_ESM.docx]

Additional files – Supplementary tables S1 and S2

Oral health related quality of life in long-term survivors of head and neck cancer compared to a general population from the seventh Tromsø study

Renate Andreassen ^1^, Birgitta Jönsson ^2,3^ and Elin Hadler-Olsen ^2,4,^*

^1^ Department of Otorhinolaryngology, University Hospital of North Norway, 9038 Tromsø, Norway

^2^ The Public Dental Health Service Competence Center of Northern Norway, 9271 Tromsø, Norway

^3^ Department of Periodontology, Institute of Odontology, The Sahlgrenska Academy at the University of Gothenburg, 41390 Gothenburg, Sweden.

^4^ Department of Medical Biology, Faculty of Health Sciences, UiT the Artic University of Norway, 9037 Tromsø, Norway

***** Correspondence: elin.hadlerolsen@tffk.no

**Table S1.** Additional treatment information Head and neck cancer cohort.

|  | **Cancer location** | | |  |
| --- | --- | --- | --- | --- |
|  | **Oral cavity** | **Pharynx** | **Larynx** |  |
| **Tooth exctraction** |  |  |  |  |
| Yes | 31 (52.5) | 60 (61.9) | 13 (25.0) |  |
| No | 28 (47.5) | 37 (38.1) | 39 (75.0) |  |
| **Tongue excisision** |  |  |  |  |
| Yes | 28 (47.5) | 8 (8.2) |  |  |
| No | 31 (52.5) | 89 (91.8) |  |  |
| **Jaw excisision** |  |  |  |  |
| Yes | 19 (32.8) |  |  |  |
| No | 39 (67.2) |  |  |  |
| **Trachestomia** |  |  |  |  |
| Yes |  | 6 (6.4) | 25 (51.0) |  |
| No |  | 79 (84.0) | 19 (38.8) |  |
| Don’t know |  | 9 (9.6) | 5 (10.2) |  |
| **Speech generator** |  |  |  |  |
| Yes |  | 1 (1.0) | 29 (56.9) |  |
| No |  | 88 (91.7) | 21 (41.2) |  |
| Don’t know |  | 7 (7.3) | 1 (2.0) |  |

**Table S2.** Oral impact on daily performances by age and gender

|  | **HNC cohort** | | **T7 cohort** | |
| --- | --- | --- | --- | --- |
|  | **No problems** | **Problems** | **No problems** | **Problems** |
| **Gender** |  |  |  |  |
| Male | 21 (17.4) | 100 (82.7) | 4568 (79.0) | 1215 (21.0) |
| Female | 13 (15.7) | 70 (84.3) | 5115 (82.0) | 1126 (18.0) |
| **Age** |  |  |  |  |
| <60 y | 2 (5.6) | 34 (94.4) | 4242 (79.4) | 1103 (20.6) |
| 60-69 y | 17 (22.1) | 60 (77.9) | 3432 (80.7) | 821 (19.3) |
| ≥ 70 y | 15 (16.5) | 76 (83.5) | 2009 (82.8) | 417 (17.2) |

HNC cohort = head and neck cancer cohort, T7 = general population cohort
